# Correlation of Redondovirus and *Entamoeba gingivalis* Detections in the Human Oral Cavity Suggests That This Amoeba Is Possibly the Redondovirus Host

**DOI:** 10.3390/ijms24076303

**Published:** 2023-03-27

**Authors:** Marine Makoa-Meng, Rayan Semmar, Angéline Antezack, Gwilherm Penant, Bernard La Scola, Virginie Monnet-Corti, Philippe Colson

**Affiliations:** 1 IHU Méditerranée Infection, 19-21 Boulevard Jean Moulin, 13005 Marseille, Francebernard.la-scola@univ-amu.fr (B.L.S.); 2 Institut de Recherche Pour le Développement (IRD), Microbes Evolution Phylogeny and Infections (MEPHI), Aix-Marseille Université, 27 Boulevard Jean Moulin, 13005 Marseille, France; 3 Ecole de Médecine Dentaire, Faculté des Sciences Médicales et Paramédicales, Aix-Marseille Université, 27 Boulevard Jean Moulin, 13385 Marseille, France; 4 Assistance Publique-Hôpitaux de Marseille (AP-HM), Hôpital Timone, Service de Parodontologie, 264 rue Saint Pierre, 13385 Marseille, France; 5 Assistance Publique-Hôpitaux de Marseille (AP-HM), 264 rue Saint-Pierre, 13005 Marseille, France

**Keywords:** redondovirus, *Entamoeba*, oral cavity, human, periodontitis

## Abstract

The virome of the human oral cavity and the relationships between viruses and diseases such as periodontitis are scarcely deciphered. Redondoviruses were reported in the human oral cavity in 2019, including in periodontitis patients. Here, we aimed at detecting redondoviruses and at searching for a potential viral host in human saliva. Non-stimulated saliva was collected between December 2020 and June 2021. These samples were tested using real-time PCR regarding the presence of redondovirus and *Entamoeba gingivalis* DNA. Similarity searches were performed using BLAST against eukaryotic and prokaryotic sequences from GenBank. The redondovirus DNA was detected in 46% of the 28 human saliva samples. In addition, short fragments of redondovirus genomes were detected in silico within *Entamoeba* sequences. Finally, *Entamoeba gingivalis* DNA was detected in 46% of the 28 saliva samples, with a strong correlation between redondovirus DNA and *E. gingivalis* DNA detections, in 93% of the cases. Regarded together, these findings and previous ones strongly support the presence of redondoviruses in the human oral cavity and their association to *E. gingivalis* as their likely host.

## 1. Introduction

The virome of the human oral cavity is currently poorly known [1,2]. This is also the case for the relationships between viruses detected at this body site and diseases such as periodontitis [3]. Periodontitis is an evolutive inflammatory disease associated with a dysbiotic microbiota, and it is characterized by the progressive destruction of dental-supporting tissues [4]. Its global prevalence has been estimated to be 20–50% among patients of any age [5,6]. Redondoviruses have been identified in 2019 in the human oral cavity including in periodontitis patients [7]. They comprise the single family that is classified in the newly established order *Recrevirales* [8] and that includes one gender, Torbevirus, divided in two species named *Brisavirus* and *Vientovirus* [9]. The genome of redondoviruses is a circular single-stranded DNA (ssDNA) of 3 kilobases (kb) with three genes including two that encode a capsid protein (Cp) and a replication-associated protein (Rep), and the ORF3 gene that encodes a protein of unknown function. Redondoviruses seem to have a global distribution as they were identified in studies from various countries [9,10,11,12,13,14,15]. In addition, they were frequently detected in the oro-respiratory tract in humans in both healthy and diseased individuals, with prevalence rates ranging between 2 and 82% [10,11,12,13,14,15]. Beyond this, they have been associated with periodontal diseases [7,11], but their prevalence in diseased patients and causative role in this disease remains controversial [7,15]. Redondovirus DNA sequences belonging to different viral genotypes were detected in the same individuals, and redondovirus DNA was also reported to persist for up to 132 days [12,15]. These viruses were also identified in the sera of pigs in Brazil, in association with respiratory symptoms [16] but not in respiratory samples from farm pigs, and in various other animals in Vietnam [15]. To date, redondoviruses have not been cultured in axenic conditions [7,12]. They have been suspected of replicating either in human cells or in eukaryotic parasites, but their host had not been identified until recently [17,18]. In our laboratory, we have started studying the viral agents of periodonditis as a complement to the analysis of the oral microbiota [19,20]. Thus, here, we aimed at detecting redondoviruses and at searching for a potential viral host in human saliva.

## 2. Results

Following the report of the presence of these viruses in the human oral cavity in correlation with periodontitis [7], we implemented qPCR systems to detect redondoviruses. We detected redondovirus DNA in 13 (46%) of the 28 human saliva samples. This high prevalence was similar to that described in Vietnam on nasal/throat swabs collected from healthy people (29 positive/58 samples; 50%) [15] or in China in gingival tissues collected from healthy people (62 positive/120 samples; 52%) [11], but was significantly higher than that reported in the United States based on metagenomic data analyses in various samples from non-diseased people in the study by Abbas et al., with a prevalence of 3.8% (45 positive/1172 samples; *p* < 10^−3^, Chi-square test) and 0.9% (2 positive/211 samples; *p* < 10^−3^, Fisher test) in the oral and nasopharyngeal samples, respectively [7].

Based on these findings, our next objective was to try determining the host of these viruses in the human oral cavity. We relied on our knowledge of the integration of viral sequences in the genomes of their hosts that is taken into account in various diagnosis settings, but also of research with virophages whose genomes can be integrated in those of their giant virus hosts or of the eukaryotes that host their associated giant viruses [21,22]. In addition, horizontal sequence transfers have been described from circular single-stranded DNA viruses to eukaryotic genomes [23]. These data led us to conduct sequence similarity searches using the BLAST tool [24] for the genomes of 52 available redondoviruses against eukaryotic and prokaryotic sequences from the GenBank nucleotide sequence database (https://www.ncbi.nlm.nih.gov/genbank/ (accessed on 1 February 2023) [25]. BLAST hits with an E-value lower than 0.01 were found for short fragments of 22 (42%) of the 52 redondovirus genomes with the genomes of *Entamoeba invadens* (Table 1; Supplementary Tables S1 and S2). *Entamoeba invadens* or *Entamoeba histolytica* sequences were the best matches in 32 cases. The mean ± standard deviation (range) size of the nucleotide alignments between the redondovirus and *Entamoeba* spp. sequences was 101 ± 54 nucleotides (42–182), while the mean BLAST score was 64.5 ± 7.8 (54.5–77.0). These were significantly higher mean values than between the redondovirus and non-*Entamoeba* spp. sequences regarding the alignment sizes (61.1 ± 9.6; *p* = 0.006) and the BLAST scores (60.1 ± 2.6; *p* = 0.03).

Using this approach, we were not able to detect similarities with sequences from *E. gingivalis* as only ribosomal gene sequences are available for this species, whereas whole genome sequences are not available [17].

Based on this finding and the fact that *E. gingivalis* was reported to be the only member of the genus *Entamoeba* that inhabits the oral cavity [25], we then searched for the presence of *E. gingivalis* in the oral cavity by implementing a qPCR system that specifically targets the 18S ribosomal gene of this amoeba. As in the study by Kinsella et al. [17], we found a very high prevalence of DNA from *E. gingivalis* in 13 (46%) of the 28 samples previously tested. In addition, we found a very strong correlation between the detections of redondovirus DNA and of *E. gingivalis* DNA, in 93% of the 28 samples, as they showed for 26 of them either the concomitant presence of DNA of these two infectious agents, in 12 cases, or their concomitant absence, in 14 cases. The redondovirus DNA was detected while *E. gingivalis* DNA was not in one case, and *E. gingivalis* DNA was detected while redondovirus DNA was not in another case (Table 2).

## 3. Discussion

We provide here data that further support the presence of redondoviruses and *E. gingivalis* in the human oral cavity and on the role of this amoeba as these viruses’ hosts. Recently, Kinsella et al. [17] reported the results of their analyses of metagenomic datasets generated from 1,124 gastrointestinal samples from seven cohorts, including 374 fecal swabs from two human cohorts and 238 oral swabs from three human cohorts, in search of sequences from Eukaryotic Circular Rep-Encoding Single-Stranded DNA (CRESS DNA) viruses. It was reported that human-associated redondoviruses were the only lineages prevalent in human oral samples, with a more sporadic presence in stool samples, which is in agreement with what had been reported by Abbas et al. in 2019 [7]. The authors, who had recently reported that two CRESS DNA virus families, *Naryaviridae* and *Nenyaviridae*, infect members of the genus *Entamoeba* (*Entamoeba histolytica*, *E. dispar*, *E. nuttalli*, or *E. invadens*) [26], then aimed to identify the potential hosts of the redondoviruses. They analyzed the ribosomal sequences generated from the different samples that were very positive for redondovirus DNA [17]. This made it to be possible to establish a short list of candidate hosts for each viral lineage and sample analyzed, based on statistical associations between the presence of the virus and that of their candidate hosts. Strikingly, a strong association was reported for the human oral samples between the presence of redondoviruses and of *E. gingivalis*. Indeed, the prevalence of redondoviruses in *E. gingivalis*-positive samples ranged between 73 and 91% compared to between 0 and 22% in *E. gingivalis*-negative samples. In addition, the normalized redondovirus loads were found to be strongly positively correlated with the *Entamoeba* loads. It should be noted that *E. gingivalis* has been reported to be the single *Entamoeba* species to inhabit the oral cavity, and its ribosomal gene sequences were those reported as the second most abundant at this human body site after human ribosomal gene sequences [27].

Another recent study, conducted by Keeler et al. [18], reported that redondoviruses are highly associated with *E. gingivalis* and may replicate within this commensal amoeba. These authors noticed that the genomes of both redondoviruses and *Entamoeba* spp. had a low GC content (approximately 34% and 24–30%, respectively), and argued that DNA virus genomes often display GC contents similar to those of their hosts genomes. Most importantly, they found a statistically significant co-occurrence of redondovirus sequences (from 81 genomes) and *E. gingivalis* sequences (from 28 genes of 18S rRNA) in metagenomes generated from the oral cavity from patients with peri-implantitis, mucositis, or periodontitis, and from healthy controls. Keeler et al. further found significant positive associations between redondovirus and *E. gingivalis* as assessed by qPCR targeting the redondovirus Cap gene or the *E. gingivalis* 18S rRNA genes in oro-/naso-pharyngeal samples and endotracheal aspirates from 38 intensive care unit patients, in respiratory samples from 88 SARS-CoV-2 patients, and in saliva from 50 healthy volunteers. In addition, Keeler et al. analyzed two metatranscriptomic datasets generated from gingival samples of periodontitis patients and healthy controls and reported that all the samples were positive for *E. gingivalis* RNA and that redondovirus RNA was only detected for diseased patients. They also performed a xenic culture of *E. gingivalis* and found it positive for redondovirus DNA and RNA. Finally, they used the chromosome conformation capture coupling with high-throughput sequencing (Hi-C) approach that can physically link DNA sequences in close vicinity [28] and were able to retrieve from the xenic culture a few chimeric sequences implicating DNA from *Entamoeba* and from redondovirus.

Regarded together, previous findings and our findings strongly support the presence of redondoviruses in the human oral cavity and their association to *E. gingivalis* as the likely host of these viruses. This widens the spectrum of viruses present in the human mouth and questions their clinical significance. Some limitations of the present work are the small number of individuals studied and the absence of a studied relationship between the detection of redondovirus and *E. gingivalis* DNA with the human oral health status. As a matter of fact, the present data combined with those from other teams prompt further investigation in future larger studies of the association between redondoviruses and *E. gingivalis*, including through performing xenic, and if possible axenic, cultures of *E. gingivalis*, and of the association between both entities and periodontitis in humans. Indeed, redondoviruses were detected in the human oral cavity from periodontitis patients [7,11,15], while *E. gingivalis* was reported to be involved in periodontal destruction and was detected in periodontal swabs from orthodontic patients [29]. In this view, it would be relevant to consider the possible interactions between redondovirus and intracellular bacteria with a sympatric lifestyle in *E. gingivalis*. Indeed, it was recently reported that a bacterial symbiont, *Parachlamydia acanthamoebae*, was capable to repress the replication of Marseillevirus in environmental isolates and laboratory strains of *Acanthamoeba*, another amoeba, which indicates that an intracellular bacterial symbiont could protect an amoebal host from viral infections [30]. Finally, other factors involved in periodontitis should also continue being studied. Among them are non-enzymatic antioxidants that notably comprise reduced glutathione, uric acid, and polyphenols and are normally supplied by the diet [31]. Indeed, their significantly lower concentrations were reported in the gingival fluid and saliva from periodontitis patients, and a reduced glutathione concentration was correlated with the degree and progression of the periodontitis [31].

## 4. Materials and Methods

### 4.1. Study Population and Sample Collection

Un-stimulated saliva (meaning individuals did not eat, drink, or brush their teeth within one hour before sample collection) samples were collected between December 2020 and June 2021, then analyzed directly or after storage at −80 °C. All the samples were anonymized. The people sampled were older than 18 years old, in good general health (according to a medical questionnaire), and in good oral health or not (gingivitis or periodontitis at any stage or grades) and were recruited by two periodontists (A.A. and V.M.C.) at the periodontal ward of university hospitals of Marseille, southern France. The only exclusion criterion was a person considered as vulnerable: pregnant woman, parturient or breast-feeding, person under guardianship or curatorship, or deprived of liberty by a judicial or administrative decision. Written consent was obtained from the sampled individuals. The study was approved by the Comité de Protection des Personnes (CPP) Sud-Ouest et Outre-Mer 1 (no. ID RCB: 2020-A01234-35-CPP 1-20-075 ID 9806).

### 4.2. Redondovirus and Entamoeba gingivalis DNA

The DNA was extracted in an 80–90 µL elution volume using the NucleoMag Pathogen kit (Macherey-Nagel, Düren, Germany) with the KingFisher Flex instrument (ThermoFisher Scientific, Waltham, MA, USA) or the EZ1 Virus mini kit v2.0 (Qiagen, Hilden, Germany) with the EZ1 Advanced XL instrument (Qiagen), and then stored at −20 °C until use. The sequences of primers and probes used for the redondovirus and *E. gingivalis* DNA are provided in Table 3.

The real-time PCR (qPCR) was performed on a Lightcycler 480 (Roche, Basel, Switzerland) or a CFX (Bio-Rad Laboratories, Inc., Hercules, CA, USA) instrument using a 20 µL reaction mixture containing 10 µL Lightcycler 480 Probe Master mix (Roche, Basel, Switzerland), 1 µL of forward primers (at a concentration of 10 µM), 0.5 µL of reverse primers (10 µM), 0.5 µL of probe (10 µM), and 5 µL of extracted DNA. A denaturation step was initiated at 95 °C for 5 min, followed by 45 cycles of 10 s at 95 °C and 30 s at 60 °C, and a final extension step of 30 s at 37 °C.

### 4.3. Detection of Eukaryotic and Prokaryotic Genomic Sequences in Redondovirus Genomes

The similarity searches were performed using the BLAST tool [24] for 52 redondovirus genomes (queries) (accession numbers MK059754-72, NC_055523, MT759843, KY244146, KY328744-46, KY579360-62, MT482428-32, MZ405019, MZ405022-23, MZ405025, MZ405030, MZ405033, MZ405035, MZ405038, MZ405041, MZ405046, MZ405050, MZ405057, MZ405062, MZ405064, MZ405066, MZ405073, MZ405075-79) against nucleotide sequences (subjects) of eukaryotes (taxid: 2759), uncultured *Eukaryota* (taxid: 100272), and *Prokaryota* (taxid: 2) members from the NCBI GenBank nucleotide database (nt) (https://www.ncbi.nlm.nih.gov/genbank/ (accessed on 1 February 2023)).

### 4.4. Statistical Analysis

The comparisons of proportions were performed using the Chi-square test and the comparisons of means were performed using the ANOVA test using the Openepi online tool (https://www.openepi.com/ (accessed on 2 February 2023)). A *p* value < 0.05 was considered statistically significant.

## Figures and Tables

**Table 1:** BLAST search results (best hits) for the 52 redondovirus genomes against eukaryotic/prokaryotic sequences from the NCBI GenBank database.

Redondovirus (Query Sequence)	Eukaryotic/Prokaryotic Species (Subject Sequence (Hit))	Position on Query	Position on Subject (Hit)	Alignment Length (Nucleotides)	BLAST Maximal Score	BLAST E-Value	Query Coverage (%)	Nucleotide Identity (%)
Vientovirus XM (MK059771.1)	*Elmis aenea* (OX393581.1)	1776–1830	27,999,280–2,799,9333	56	59.9	4.0 × 10^−3^	1	87.5
	*Caradrina clavipalpis* (OW052103.1)	1776–1818	4,879,153–4,879,194	43	57.2	1.5 × 10^−2^	1	82.3
	*Vespula germanica* (HG996531.1)	1777–1818	8,520,621–8,520,663	43	57.2	1.5 × 10^−2^	1	81.0
	*Entamoeba histolytica* (AP023130.1)	986–1027	463,656–463,697	42	54.5	1.8 × 10^−1^	1	88.1
Vientovirus MW (MK059772.1)	*Entamoeba invadens* (XM_004185684.1)	2214–2301	661–773	89	59	4.0 × 10^−3^	2	75.3
	*Entamoeba histolytica* (AP023130.1)	986–1031	463,656–463,701	46	57.2	1.5 × 10^−2^	1	87.0
Vientovirus MC (MK059770.1)	*Chrysoperla carnea* (FR997758.1)	1770–1837	72,667,248–72,667,313	68	58.1	1.5 × 10^−2^	2	79.4
	*Entamoeba histolytica* (AP023130.1)	986–1027	463,656–463,697	42	54.5	1.8 × 10^−1^	1	88.1
Vientovirus LZ (MK059769.1)	*Entamoeba invadens* (XM_004185684.1)	2210–2294	678–762	86	72.5	7.0 × 10^−7^	2	80.2
	*Entamoeba histolytica* (AP023130.1)	986–1031	463,656–463,701	46	57.2	1.5 × 10^−2^	1	87.0
Vientovirus EC (MK059768.1)	*Agriphila geniculea* (OX038883.1)	1768–1822	12,573,469–12,573,521	55	61.7	1.0 × 10^−3^	1	85.5
	*Clistopyga incitator* (OX382180.1)	1772–1826	8,425,204–8,425,260	57	60.8	1.0 × 10^−3^	1	86.0
	*Agonopterix arenella* (OV656709.1)	1796–1832	14,531,762–14,531,798	37	59	4.0 × 10^−3^	1	94.6
	*Entamoeba histolytica* (AP023130.1)	986–1031	463,656–463,701	46	57.2	1.5 × 10^−2^	1	87.0
Vientovirus AV (MK059767.1)	*Entamoeba histolytica* (AP023130.1)	986–1031	463,656–463,701	46	57.2	1.5 × 10^−2^	1	87.0
Vientovirus LT ((MK059766.1)	*Caradrina kadenii* (OX381679.1)	1275–1329	12,497,450–12,497,508	59	57.2	1.5 × 10^−2^	1	83.1
Vientovirus FB (MK059763.1)	*Entamoeba invadens* (XM_004185684.1)	2125–2297	673–843	174	70.7	2.0 × 10^−6^	5	70.1
	*Entamoeba histolytica* (AP023130.1)	986–1031	463,656–463,701	46	57.2	1.5 × 10^−2^	1	87.0
Vientovirus ES (MK059762.1)	*Spiroplasma turonicum* (CP013860.1)	2449–2531	840,669–840,750	83	61.7	1.0 × 10^−3^	2	77.1
	*Allantophomopsis cytisporea* (CP103029.1)	2888–2936	1,082,977–1,083,025	49	58.1	1.0 × 10^−2^	1	85.7
	*Entamoeba histolytica* (AP023130.1)	986–1031	463,656–463,701	46	57.2	1.5 × 10^−2^	1	87.0
Vientovirus DC (MK059761.1)	*Entamoeba invadens* (XM_004185684.1)	2122–2294	673–843	174	70.7	2.0 × 10^−6^	5	70.1
	*Entamoeba histolytica* (AP023130.1)	986–1031	463,656–463,701	46	57.2	1.5 × 10^−2^	1	87.0
Vientovirus AL (MK059760.1)	No hit	No hit	No hit	No hit	No hit	No hit	–	–
Vientovirus VN (MT759843.1)	*Entamoeba invadens* (XM_004185684.1)	2207–2284	685–762	79	59.9	4.0 × 10^−3^	2	78.5
	*Boloria selene* (HG993153.1)	2937–3001	6,447,257–6,447,322	66	58.1	1.5 × 10^−2^	2	80.3
	*Entamoeba histolytica* (AP023130.1)	986–1031	463,656–463,701	46	57.2	1.5 × 10^−2^	1	87.0
Vientovirus JB (MK059764.1)	*Allantophomopsis cytisporea* (CP103029.1)	2884–2942	1,082,977–1,083,034	59	59	4.0 × 10^−3^	1	83.1
	*Iphiclides podalirius* (OW152837.1)	1782–1843	3,451,464–3,451,522	62	57.2	1.5 × 10^−2^	2	82.3
	*Entamoeba histolytica* (AP023130.1)	986–1031	463,656–463,701	46	57.2	1.5 × 10^−2^	1	87.0
Vientovirus JY (MK059765.1)	*Allantophomopsis cytisporea* (CP103029.1)	2885–2933	1,082,977–1,083,025	49	58.1	1.5 × 10^−2^	1	85.7
Brisavirus II (MK059755.1)	*Lymantria monacha* (LR991098.1)	1804–1881	24,094,134–24,094,211	78	60.8	1.0 × 10^−3^	2	76.9
	*Myopa tessellatipennis* (OX031314.1)	1828–1866	67,193,903–67,193,941	39	58.1	1.5 × 10^−2^	1	92.3
	*Abrostola triplasia* (OX276447.1)	1764–1854	919,903–919,988	91	57.2	1.5 × 10^−2^	3	74.7
	*Entamoeba histolytica* (AP023130.1)	986–1031	463,656–463,701	46	57.2	1.5 × 10^−2^	1	87.0
Brisavirus MD (MK059756.1)	*Micropterix aruncella (OX155967.1)*	1111–1167	11,485,724–11,485,780	58	58.1	1.5 × 10^−2^	1	84.5
Brisavirus VW (MK059759.1)	*Ananas comosus (LR862130.1)*	2453–2508	1,939,365–1,939,420	56	66.2	3.0 × 10^−5^	1	85.7
	*Amphipyra berbera (OU343149.1)*	1773–1824	6,458,724–6,458,776	53	57.2	1.4 × 10^−2^	1	84.9
Brisavirus RC (MK059757.1)	*Lochmaea capreae* (OX421399.1)	2504–2577	6,703,802–6,703,876	75	65.3	1.0 × 10^−4^	2	80.0
	*Teleiodes luculella* (OX419593.1)	2430–2510	1,146,107–1,146,186	82	57.2	1.5 × 10^−2^	2	78.1
	*Entamoeba histolytica* (AP023130.1)	986–1027	463,656–463,697	42	54.5	1.8 × 10^−1^	1	88.1
Brisavirus YH (MK059758.1)	*Entamoeba invadens* (XM_004185684.1)	2061–2242	688–866	182	61.7	1.0 × 10^−3^	5	68.1
	*Entamoeba histolytica* (AP023130.1)	986–1031	463,656–463,701	46	57.2	1.5 × 10^−2^	1	87.0
Brisavirus AA (MK059754.1)	*Entamoeba histolytica* (AP023130.1)	986–1031	463,656–463,701	46	57.2	1.5 × 10^−2^	1	87.0
Human respiratory circular DNA virus isolate 15232 (KY328746.1)	*Entamoeba invadens* (XM_004185684.1)	2211–2295	678–762	86	72.5	7.0 × 10^−7^	2	80.2
	*Entamoeba histolytica* (AP023130.1)	986–1031	463,656–463,701	46	57.2	1.5 × 10^−2^	1	87.0
Human respiratory circular DNA virus isolate 15037 (KY328745.1)	*Entamoeba invadens* (XM_004185684.1)	2211–2295	678–762	86	77	2.0 × 10^−8^	2	81.4
	*Entamoeba histolytica* (AP023130.1)	986–1031	463,656–463,701	46	57.2	1.5 × 10^−2^	1	87.0
Human respiratory circular DNA virus isolate 15040 (KY244146.1), 15065 (KY579361.1), 15027 (KY579360.1), 15078 (KY579362.1)	*Entamoeba invadens* (XM_004185684.1)	2211–2295	678–762	86	72.5	7.0 × 10^−7^	2	80.2
	*Entamoeba histolytica* (AP023130.1)	986–1031	463,656–463,701	46	57.2	1.0 × 10^−2^	1	87.0
Redondovirus sp. isolate 1 (MT482428.1)	*Entamoeba histolytica* (AP023130.1)	989–1030	463,656–463,697	42	54.5	1.8 × 10^−1^	1	88.1
Redondovirus sp. isolate 10 (MT482429.1)	*Entamoeba invadens* (XM_004185684.1)	2203–2277	681–755	75	55.4	5.1 × 10^−2^	2	76.0
	*Entamoeba histolytica* (AP023130.1)	986–1027	463,656–463,697	42	54.5	1.8 × 10^−1^	1	88.1
Redondovirus sp. isolate 11 (MT482430.1)	*Entamoeba invadens* (XM_004185684.1)	2125–2297	673–843	174	75.2	5.0 × 10^−8^	5	70.7
	*Entamoeba histolytica* (AP023130.1)	986–1031	463,656–463,701	46	57.2	1.5 × 10^−2^	1	87.0
Redondovirus sp. isolate 25 (MT482431.1)	*Entamoeba histolytica* (AP023130.1)	986–1027	463,656–463,697	42	54.5	1.8 × 10^−1^	1	88.1
Redondovirus sp. isolate 26 (MT482432.1)	*Entamoeba histolytica* (AP023130.1)	986–1027	463,656–463,697	42	54.5	1.8 × 10^−1^	1	88.1
Vientovirus isolate p67_20161228_ET_WGA_B (MZ405079.1)	*Oppiella nova* (OC947375.1)	2921–2975	562–614	57	59.9	4.0 × 10^−3^	1	86.0
	*Entamoeba histolytica* (AP023130.1)	986–1031	463,656–463,701	46	57.2	1.5 × 10^−2^	1	87.0
Vientovirus isolate p67_20161228_ET_WGA_A (MZ405078.1)	*Entamoeba invadens* (XM_004185684.1)	2208–2292	678–762	86	72.5	7.0 × 10^−7^	2	80.2
	*Oppiella nova* (OC947375.1)	2922–2976	562–614	57	59.9	4.0 × 10^−3^	1	86.0
	*Entamoeba histolytica* (AP023130.1)	986–1031	463,656–463,701	46	57.2	1.5 × 10^−2^	1	87.0
Vientovirus isolate p67_20161223_ET_A1 (MZ405077.1)	*Entamoeba histolytica* (AP023130.1)	986–1031	463,656–463,701	46	57.2	1.5 × 10^−2^	1	87.0
Vientovirus isolate p67_20161216_ET_C1 (MZ405076.1)	*Oppiella nova* (OC947375.1)	2921–2975	562–614	57	59.9	4.0 × 10^−3^	1	86.0
	*Entamoeba histolytica* (AP023130.1)	986–1031	463,656–463,701	46	57.2	1.5 × 10^−2^	1	87.0
Vientovirus isolate p67_20161216_ET_B10 (MZ405075.1)	*Oppiella nova* (OC947375.1)	2918–2972	562–614	57	59.9	4.0 × 10^−3^	1	86.0
	*Entamoeba histolytica* (AP023130.1)	986–1031	463,656–463,701	46	57.2	1.5 × 10^−2^	1	87.0
Brisavirus isolate p67_20161208_OP_D7 (MZ405073.1)	*Phlogophora meticulosa* (LR990517.1)	317–370	12,271,425–12,271,478	55	57.2	1.5 × 10^−2^	1	85.5
	*Entamoeba histolytica* (AP023130.1)	986–1031	463,656–463,701	46	57.2	1.5 × 10^−2^	1	87.0
Brisavirus isolate p48_v2_ET_w8_c3 (MZ405057.1)	*Entamoeba invadens* (XM_004185684.1)	2069–2250	688–866	182	66.2	3.0 × 10^−5^	5	68.7
	*Entamoeba histolytica* (AP023130.1)	986–1027	463,656–463,697	42	54.5	1.8 × 10^−1^	1	88.1
Vientovirus isolate p48_v2_ET_w11_c2 (MZ405066.1)	*Entamoeba invadens* (XM_004185684.1)	2125–2297	673–843	174	70.7	2.0 × 10^−6^	5	70.1
	*Entamoeba histolytica* (AP023130.1)	986–1031	463,656–463,701	46	57.2	1.5 × 10^−2^	1	87.0
Vientovirus isolate p48_v2_ET_w10_c4 (MZ405064.1)	*Entamoeba invadens* (XM_004185684.1)	2125–2297	673–843	174	70.7	2.0 × 10^−6^	5	70.1
	*Entamoeba histolytica* (AP023130.1)	986–1031	463,656–463,701	46	57.2	1.5 × 10^−2^	1	87.0
Vientovirus isolate p48_v2_ET_w9_c4 (MZ405062.1)	*Entamoeba invadens* (XM_004185684.1)	2125–2297	673–843	174	70.7	2.0 × 10^−6^	5	70.1
	*Entamoeba histolytica* (AP023130.1)	986–1031	463,656–463,701	46	57.2	1.5 × 10^−2^	1	87.0
Vientovirus isolate p48_v1_ET_w5_c4 (MZ405050.1)	*Entamoeba invadens* (XM_004185684.1)	2212–2289	685–762	79	59.9	4.0 × 10^−3^	2	78.5
	*Entamoeba histolytica* (AP023130.1)	986–1031	463,656–463,701	46	57.2	1.5 × 10^−2^	1	87.0
Vientovirus isolate p48_v1_ET_w4_c3 (MZ405046.1)	*Entamoeba invadens* (XM_004185684.1)	2125–2297	673–843	174	70.7	2.0 × 10^−6^	5	70.1
	*Entamoeba histolytica* (AP023130.1)	986–1031	463,656–463,701	46	57.2	1.5 × 10^−2^	1	87.0
Vientovirus isolate p48_v1_ET_w2_c2 (MZ405041.1)	*Entamoeba invadens* (XM_004185684.1)	2125–2297	673–843	174	70.7	2.0 × 10^−6^	5	70.1
	*Entamoeba histolytica* (AP023130.1)	986–1031	463,656–463,701	46	57.2	1.5 × 10^−2^	1	87.0
Vientovirus isolate p48_v1_ET_w1_c4 (MZ405038.1)	*Entamoeba invadens* (XM_004185684.1)	2212–2289	685–762	79	59.9	4.0 × 10^−3^	2	78.5
	*Entamoeba histolytica* (AP023130.1)	986–1031	463,656–463,701	46	57.2	1.5 × 10^−2^	1	87.0
Vientovirus isolate ET738–12 (MZ405035.1)	*Entamoeba invadens* (XM_004185684.1)	2212–2289	685–762	79	59.9	4.0 × 10^−3^	2	78.5
	*Entamoeba histolytica* (AP023130.1)	986–1031	463,656–463,701	46	57.2	1.5 × 10^−2^	1	87.0
Vientovirus isolate ET203–9 (MZ405022.1)	*Entamoeba histolytica* (AP023130.1)	986–1027	463,656–463,697	42	54.5	1.8 × 10^−1^	1	88.1
Vientovirus isolate ET724–8 (MZ405033.1)	*Entamoeba invadens* (XM_004185684.1)	2212–2289	685–762	79	72.5	7.0 × 10^−7^	2	80.2
	*Entamoeba histolytica* (AP023130.1)	986–1027	463,656–463,697	42	54.5	1.8 × 10^−1^	1	88.1
Vientovirus isolate ET724–2 (MZ405030.1)	*Entamoeba invadens* (XM_004185684.1)	2194–2278	678–762	86	72.5	7.0 × 10^−7^	2	80.2
	*Chrysoperla carnea* (FR997758.1)	1771–1837	72,667,248–72,667,313	67	59.9	4.0 × 10^−3^	2	80.6
	*Entamoeba histolytica* (AP023130.1)	986–1027	463,656–463,697	42	54.5	1.8 × 10^−1^	1	88.1
Vientovirus isolate ET207–1 (MZ405023.1)	*Entamoeba histolytica* (AP023130.1)	986–1027	463,656–463,697	42	54.5	1.8 × 10^−1^	1	88.1
Vientovirus isolate CM895–9 (MZ405019.1)	*Entamoeba histolytica* (AP023130.1)	986–1027	463,656–463,697	42	54.5	1.8 × 10^−1^	1	88.1
Brisavirus isolate ET239–2 (MZ405025.1)	*Gossypium turneri* (CP032573.1)	1795–1848	4,332,814–4,332,869	56	59	4.0 × 10^−3^	1	83.9
	*Clostridium perfringens* (LR607381.1)	2443–2502	2,174,153–2,174,213	61	58.1	1.5 × 10^−2^	1	82.0
	*Pherbina coryleti* (OX030953.1)	1785–1846	1,527,490–1,527,547	63	57.2	1.5 × 10^−2^	2	82.5

*Entamoeba* spp. genomes are highlighted with a light grey background. NCBI GenBank database: https://www.ncbi.nlm.nih.gov/genbank/ (accessed on 1 February 2023) [25].

**Table 2:** Correlation between redondovirus and *Entamoeba gingivalis* DNA detection using real-time PCR.

		Redondovirus DNA Detection Using qPCR	
		Positive	Negative
*Entamoeba gingivalis* DNA detection using qPCR	Positive	12	1
	Negative	1	14

This table correlates the results of qPCR detection of redondovirus DNA (positive or negative qPCR detection) and *Entamoeba gingivalis* DNA (positive or negative qPCR detection).

**Table 3:** Primer and probe sequences used for redondovirus and *E. gingivalis* DNA detection using real-time PCR.

Primer/Probe Name	Sequence (5′-3′)	Targeted Gene and Coordinates (Nucleotides ^a^)	PCR Product Size (Base Pairs)
Pan-Redondo-Cp-Fwd (forward primer)	TAATGATGCTCTTAATCARTATG	Capsid gene (1443–1465)	53
Pan-HCRV-AA-Rev [7] (reverse primer)	CTCGAAATCTTCCTATACTGGTAT	Capsid gene (1518–1541)	
Pan-HCRV-AA-Probe [7] (probe)	AAATGGAAGGGAGAGAGGCCTTTGG	Capsid gene (1492–1516)	
Redondo-Cp-2F (forward primer)	CTAAGMGATATGCATCAAGAAAGAG	Capsid gene (5–29)	162
Redondo-Cp-2R (reverse primer)	CTGGCAAAGGTGTTAAGAATAAAT	Capsid gene (191–214)	
Redondo-Cp-2P (probe)	AAGAAGATTAGAAGGGCTAAAAGGCAATATAA	Capsid p gene (151–182)	
*E. gingivalis*-F (forward primer)	GAATCAATGARAATATCTGATCTATC	18S rRNA gene (115–140)	145
*E. gingivalis*-R (reverse primer)	GGTAGTGACGACAAATAACTCTATT	18S rRNA gene (285–309)	
*E. gingivalis*-P (probe)	AATTAGGGTTTGACATCGGAGAAG	18S rRNA gene (190–213)	

^a^ Relative to genomes GenBank Accession no. NC_055523 (redondovirus) and KX027286 (*E. gingivalis*). rRNA, ribosomal RNA.

## Data Availability

Not applicable.

## Supplementary Materials

The following supporting information can be downloaded at: https://www.mdpi.com/article/10.3390/ijms24076303/s1.

## References

[1] Martínez A., Kuraji R., Kapila Y.L. (2021). The human oral virome: Shedding light on the dark matter. Periodontol 2000.

[2] Liang G., Bushman F.D. (2021). The human virome: Assembly, composition and host interactions. Nat. Rev. Microbiol..

[3] Aggarwal T., Lamba A.K., Faraz F., Tandon S. (2017). Viruses: Bystanders of periodontal disease. Microb. Pathog..

[4] Williams R.C. (1990). Periodontal disease. N. Engl. J. Med..

[5] Albandar J.M., Rams T.E. (2002). Global epidemiology of periodontal diseases: An overview. Periodontol 2000.

[6] Eke P.I., Thornton-Evans G.O., Wei L., Borgnakke W.S., Dye B.A., Genco R.J. (2018). Periodontitis in US Adults: National Health and Nutrition Examination Survey 2009-2014. J. Am. Dent. Assoc..

[7] Abbas A.A., Taylor L.J., Dothard M.I., Leiby J.S., Fitzgerald A.S., Khatib L.A., Collman R.G., Bushman F.D. (2019). Redondoviridae, a Family of Small, Circular DNA Viruses of the Human Oro-Respiratory Tract Associated with Periodontitis and Critical Illness. Cell Host Microbe.

[8] Krupovic M., Varsani A., Kazlauskas D., Breitbart M., Delwart E., Rosario K., Yutin N., Wolf Y.I., Harrach B., Zerbini F.M. (2020). Cressdnaviricota: A Virus Phylum Unifying Seven Families of Rep-Encoding Viruses with Sin-gle-Stranded, Circular DNA Genomes. J. Virol..

[9] Abbas A., Taylor L.J., Collman R.G., Bushman F.D. (2021). Ictv Report Consortium. ICTV Virus Taxonomy Profile: Redondoviridae. J. Gen. Virol..

[10] Cui L., Wu B., Zhu X., Guo X., Ge Y., Zhao K., Qi X., Shi Z., Zhu F., Sun L. (2017). Identification and genetic characteriza-tion of a novel circular single-stranded DNA virus in a human upper respiratory tract sample. Arch. Virol..

[11] Zhang Y., Wang C., Feng X., Chen X., Zhang W. (2021). Redondoviridae and periodontitis: A case-control study and identification of five novel redondoviruses from periodontal tissues. Virus Evol..

[12] Taylor L.J., Dothard M.I., Rubel M.A., Allen A.A., Hwang Y., Roche A.M., Graham-Wooten J., Fitzgerald A.S., Khatib L.A., Ranciaro A. (2021). Redondovirus Diversity and Evolution on Global, Individual, and Molecular Scales. J. Virol..

[13] Lázaro-Perona F., Dahdouh E., Román-Soto S., Jiménez-Rodríguez S., Rodríguez-Antolín C., de la Calle F., Agrifoglio A., Membrillo F.J., García-Rodríguez J., Mingorance J. (2020). Metagenomic Detection of Two Vientoviruses in a Human Sputum Sample. Viruses.

[14] Spezia P.G., Macera L., Mazzetti P., Curcio M., Biagini C., Sciandra I., Turriziani O., Lai M., Antonelli G., Pistello M. (2020). Redondovirus DNA in human respiratory samples. J. Clin. Virol..

[15] Tu N.T.K., Deng X., Hong N.T.T., Ny N.T.H., Phuc T.M., Tam P.T.T., Han D.A., Ha L.T.T., Thwaites G., Doorn H.R.V. (2021). Redondoviridae: High Prevalence and Possibly Chronic Shedding in Human Respiratory Tract, But No Zoonotic Transmission. Viruses.

[16] Tochetto C., Cibulski S.P., Muterle Varela A.P., Cerva C., Alves de Lima D., Fumaco Teixeira T., Quoos Mayer F., Roehe P.M. (2021). A variety of highly divergent eukaryotic ssDNA viruses in sera of pigs. J. Gen. Virol..

[17] Kinsella C.M., Deijs M., Becker C., Broekhuizen P., van Gool T., Bart A., Schaefer A.S., van der Hoek L. (2022). Host prediction for disease-associated gastrointestinal cressdnaviruses. Virus Evol..

[18] Keeler E.L., Merenstein C., Reddy S., Taylor L.J., Cobián-Güemes A.G., Zankharia U., Collman R.G., Bushman F.D. (2023). Widespread, human-associated redondoviruses infect the commensal protozoan Entamoeba gingivalis. Cell Host Microbe.

[19] Antezack A., Boxberger M., Ben Khedher M., La Scola B., Monnet-Corti V. (2021). Isolation and description of *Selenomonas timonae* sp. nov., a novel *Selenomonas* species detected in a gingivitis patient. Int. J. Syst. Evol. Microbiol..

[20] Antezack A., Boxberger M., Rolland C., Monnet-Corti V., La Scola B. (2021). Isolation and Characterization of *Kingella bonacorsii* sp. nov., A Novel *Kingella* Species Detected in a Stable Periodontitis Subject. Pathogens.

[21] La Scola B., Desnues C., Pagnier I., Robert C., Barrassi L., Fournous G., Merchat M., Suzan-Monti M., Forterre P., Koonin E. (2008). The virophage as a unique parasite of the giant mimivirus. Nature.

[22] Fischer M.G., Hackl T. (2016). Host genome integration and giant virus-induced reactivation of the virophage mavirus. Nature.

[23] Liu H., Fu Y., Li B., Yu X., Xie J., Cheng J., Ghabrial S.A., Li G., Yi X., Jiang D. (2011). Widespread horizontal gene transfer from circular single-stranded DNA viruses to eukaryotic genomes. BMC Evol. Biol..

[24] Altschul S.F., Gish W., Miller W., Myers E.W., Lipman D.J. (1990). Basic local alignment search tool. J. Mol. Biol..

[25] Sayers E.W., Cavanaugh M., Clark K., Pruitt K.D., Schoch C.L., Sherry S.T., Karsch-Mizrachi I. (2022). GenBank. Nucleic Acids Res..

[26] Kinsella C.M., Bart A., Deijs M., Broekhuizen P., Kaczorowska J., Jebbink M.F., van Gool T., Cotton M., van der Hoek L. (2020). Entamoeba and Giardia parasites implicated as hosts of CRESS viruses. Nat. Commun..

[27] Deng Z.L., Szafrański S.P., Jarek M., Bhuju S., Wagner-Döbler I. (2017). Dysbiosis in chronic periodontitis: Key microbial players and interactions with the human host. Sci. Rep..

[28] Lu L., Jin F. (2023). Easy Hi-C: A Low-Input Method for Capturing Genome Organization. Methods Mol. Biol..

[29] Contaldo M., Lucchese A., Lajolo C., Rupe C., Di Stasio D., Romano A., Petruzzi M., Serpico R. (2021). The Oral Microbiota Changes in Orthodontic Patients and Effects on Oral Health: An Overview. J. Clin. Med..

[30] Arthofer P., Delafont V., Willemsen A., Panhölzl F., Horn M. (2022). Defensive symbiosis against giant viruses in amoebae. Proc. Natl. Acad. Sci. USA.

[31] Toczewska J., Maciejczyk M., Zalewska A., Konopka T. (2022). Gingival fluid and saliva concentrations of selected non-enzymatic antioxidants in periodontitis. Dent. Med. Probl..

