# Supplementary material for: Correlation of Redondovirus and Entamoeba gingivalis Detections in the Human Oral Cavity Suggests That This Amoeba Is Possibly the Redondovirus Host
Source: Int J Mol Sci. 2023 Mar 27;24(7):6303. doi: 10.3390/ijms24076303 (PMC10094137; doi:10.3390/ijms24076303)
Supplement: Supplementary file 1 [file ijms-24-06303-s001.zip › ijms-2244532-supplementary.pdf]

| Redondovirus genomes (query sequences) | Position on query | Query sequence fragment aligned with *Entamoeba* spp. DNA | Species hit |
|---|---|---|---|
| Vientovirus MW (MK059772.1) | 2214-2301 | CTGAAATCGTCAATGATTGCAACTGGTTGACCTCTATAGCCGTCGAACCATTTAAGTGTTCATTTGACATCCAGTATTCACCATTAA | Entamoeba invadens (XM_004185684.1) |
| Vientovirus MW (MK059772.1) | 986-1031 | ATAAAGTATATGATATGTCAAAGCCTTTTTCATTCTTTGTTAGGCC | Entamoeba histolytica (AP023130.1) |
| Vientovirus XM (MK059771.1) | 986-1027 | ATAAAGTATATGATATGTCAAAGCCTTTTTCATTCTTTGTTA | Entamoeba histolytica (AP023130.1) |
| Vientovirus MC (MK059770.1) | 986-1027 | ATAAAGTATATGATATGTCAAAGCCTTTTTCATTCTTTGTTA | Entamoeba histolytica (AP023130.1) |
| Vientovirus LZ (MK059769.1) | 2210-2294 | TCTGAAGTCATCAATTATTGCGAGCTTTTGACCGTGATATCCATCAAACCAACGTAAGGAGTCGTTTGATATCCAATAGTCTCCA | *Entamoeba invadens* (XM_004185684.1) |
| Vientovirus LZ (MK059769.1) | 986-1031 | ATAAAGTATATGATATGTCAAAGCCTTTTTCATTCTTTGTTAGGCC | Entamoeba histolytica (AP023130.1) |
| Vientovirus EC (MK059768.1) | 986-1031 | ATAAAGTATATGATATGTCAAAGCCTTTTTCATTCTTTGTTAGGCC | Entamoeba histolytica (AP023130.1) |
| Vientovirus AV (MK059767.1) | 986-1031 | ATAAAGTATATGATATGTCAAAGCCTTTTTCATTCTTTGTTAGGCC | Entamoeba histolytica (AP023130.1) |
| Vientovirus FB (MK059763.1) | 2125-2297 | TACAAATCCTCCTTTCACTTGTACGATCAGGTTATAGCCATCAAGTAACCTGAGGAGGAAGCTCCATTCTGTAAGCATCGATTTCCTGAAGTCGTCAATGATTG | Entamoeba invadens (XM_004185684.1) |
| Vientovirus FB (MK059763.1) | 986-1031 | ATAAAGTATATGATATGTCAAAGCCTTTTTCATTCTTTGTTAGGCC | Entamoeba histolytica (AP023130.1) |
| Vientovirus ES (MK059762.1) | 986-1031 | ATAAAGTATATGATATGTCAAAGCCTTTTTCATTCTTTGTTAGGCC | Entamoeba histolytica (AP023130.1) |
| Vientovirus DC (MK059761.1) | 2122-2294 | TACAAATCCTCCTTTCACTTGTACGATCAGGTTATAGCCATCAAGTAACCTGAGGAGGAAGCTCCATTCTGTAAGCATCGATTTCCTGAAGTCGTCAATGATTG | Entamoeba invadens (XM_004185684.1) |
| Vientovirus DC (MK059761.1) | 986-1031 | ATAAAGTATATGATATGTCAAAGCCTTTTTCATTCTTTGTTAGGCC | Entamoeba histolytica (AP023130.1) |
| Vientovirus VN (MT759843.1) | 2207-2284 | TCTGAAGTCATCAATGATGGCGATTTCTTGACCGTTGTAATTATCAAACCACTTTAAGGTTTCGTTCGAGATCCAATA | Entamoeba invadens (XM_004185684.1) |
| Vientovirus VN (MT759843.1) | 986-1031 | ATAAAGTATATGATATGTCAAAGCCTTTTTCATTCTTTGTTAGGCC | Entamoeba histolytica (AP023130.1) |
| Vientovirus JB (MK059764.1) | 986-1031 | ATAAAGTATATGATATGTCAAAGCCTTTTTCATTCTTTGTTAGGCC | Entamoeba histolytica (AP023130.1) |
| Brisavirus II (MK059755.1) | 986-1031 | ATAAAGTATATGATATGTCAAAGCCTTTTTCATTCTTTGTTAGGCC | Entamoeba histolytica (AP023130.1) |
| Brisavirus RC (MK059757.1) | 986-1027 | ATAAAGTATATGATATGTCAAAGCCTTTTTCATTCTTTGTTA | Entamoeba histolytica (AP023130.1) |
| Brisavirus YH (MK059758.1) | 2061-2242 | ATTATTATCTTTGGTTCCCATTTTACGAACCCTCCCTTTATTTGCACAATGAGGGGATATCTGTCGAGTAAGCGTAACAAGAATGCCCATGTAGGCAATATATC TCTTCTTAAATCATCAAAGACAGCTATTTCTTGTCCACAATAGCCATCAAACCATTTCAGGCTATCGTTCGAGATCCA | Entamoeba invadens (XM_004185684.1) |
| Brisavirus AA (MK059754.1) | 986-1031 | ATAAAGTATATGATATGTCAAAGCCTTTTTCATTCTTTGTTAGGCC | Entamoeba histolytica (AP023130.1) |
| Human respiratory circular DNA virus isolate 15232 (KY328746.1) | 2211-2295 | TCTGAAGTCATCAATTATTGCGAGCTTTTGACCGTGATATCCATCAAACCAACGTAAGGAGTCGTTTGATATCCAATAGTCTCCA | Entamoeba invadens (XM_004185684.1) |
| Human respiratory circular DNA virus isolate 15232 (KY328746.1) | 986-1031 | ATAAAGTATATGATATGTCAAAGCCTTTTTCATTCTTTGTTAGGCC | Entamoeba histolytica (AP023130.1) |
| Human respiratory circular DNA virus isolate 15037 (KY328745.1) | 2211-2295 | TCTGAAGTCATCAATTATTGCGAGCTTTTGACCGTGATATCCATCAAACCAACGTAAGGAGTCGTTTGATATCCAATAGTCTCCA | Entamoeba invadens (XM_004185684.1) |
| Human respiratory circular DNA virus isolate 15037 (KY328745.1) | 986-1031 | ATAAAGTATATGATATGTCAAAGCCTTTTTCATTCTTTGTTAGGCC | Entamoeba histolytica (AP023130.1) |
| Human respiratory circular DNA virus isolate 15040 (KY244146.1), 15065 (KY579361.1), 15027 (KY579360.1), 15078 (KY579362.1) | 2211-2295 | TCTGAAGTCATCAATTATTGCGAGCTTTTGACCGTGATATCCATCAAACCAACGTAAGGAGTCGTTTGATATCCAATAGTCTCCA | Entamoeba invadens (XM_004185684.1) |
| Human respiratory circular DNA virus isolate 15040 (KY244146.1), 15065 (KY579361.1), 15027 (KY579360.1), 15078 (KY579362.1) | 986-1031 | ATAAAGTATATGATATGTCAAAGCCTTTTTCATTCTTTGTTAGGCC | Entamoeba histolytica (AP023130.1) |
| Redondovirus sp. Isolate 1 (MT482428.1) | 989-1030 | ATAAAGTATATGATATGTCAAAGCCTTTTTCATTCTTTGTTA | Entamoeba histolytica (AP023130.1) |
| Redondovirus sp. Isolate 10 (MT482429.1) | 2203-2277 | TCGTCTATTATAGCAACTTTCTGGCTATTATAGCCGTCGAACCAGTGAAGATTGTCATTCGTTATCCAATAGTCT | Entamoeba invadens (XM_004185684.1) |
| Redondovirus sp. Isolate 10 (MT482429.1) | 986-1027 | ATAAAGTATATGATATGTCAAAGCCTTTTTCATTCTTTGTTA | Entamoeba histolytica (AP023130.1) |
| Redondovirus sp. Isolate 11 (MT482430.1) | 2125-2297 | TACAAATCCTCCTTTCACTTGAACGATCAGGTTATAGCCATCAAGTAACCTGAGGAGGAAGCTCCATTCTGTAAGCATCGATTTCCTGAAGTCGTCAATGATTG | Entamoeba invadens (XM_004185684.1) |
| Redondovirus sp. Isolate 11 (MT482430.1) | 986-1031 | ATAAAGTATATGATATGTCAAAGCCTTTTTCATTCTTTGTTAGGCC | Entamoeba histolytica (AP023130.1) |
| Redondovirus sp. Isolate 25 (MT482431.1) | 986-1027 | ATAAAGTATATGATATGTCAAAGCCTTTTTCATTCTTTGTTA | Entamoeba histolytica (AP023130.1) |
| Redondovirus sp. Isolate 26 (MT482432.1) | 986-1027 | ATAAAGTATATGATATGTCAAAGCCTTTTTCATTCTTTGTTA | Entamoeba histolytica (AP023130.1) |
| Vientovirus isolate p67_20161228_ET_WGA_B (MZ405079.1) | 986-1031 | ATAAAGTATATGATATGTCAAAGCCTTTTTCATTCTTTGTTAGGCC | Entamoeba histolytica (AP023130.1) |
| Vientovirus isolate p67_20161228_ET_WGA_A (MZ405078.1) | 2208-2292 | TCTGAAGTCATCAATTATTGCGAGCTTTTGACCGTGATATCCATCAAACCAACGTAAGGAGTCGTTTGATATCCAATAGTCTCCA | Entamoeba invadens (XM_004185684.1) |
| Vientovirus isolate p67_20161228_ET_WGA_A (MZ405078.1) | 986-1031 | ATAAAGTATATGATATGTCAAAGCCTTTTTCATTCTTTGTTAGGCC | Entamoeba histolytica (AP023130.1) |
| Vientovirus isolate p67_20161223_ET_A1 (MZ405077.1) | 986-1031 | ATAAAGTATATGATATGTCAAAGCCTTTTTCATTCTTTGTTAGGCC | Entamoeba histolytica (AP023130.1) |
| Vientovirus isolate p67_20161216_ET_C1 (MZ405076.1) | 986-1031 | ATAAAGTATATGATATGTCAAAGCCTTTTTCATTCTTTGTTAGGCC | Entamoeba histolytica (AP023130.1) |
| Vientovirus isolate p67_20161216_ET_B10 (MZ405075.1) | 986-1031 | ATAAAGTATATGATATGTCAAAGCCTTTTTCATTCTTTGTTAGGCC | Entamoeba histolytica (AP023130.1) |
| Brisavirus isolate p67_20161208_OP_D7 (MZ405073.1) | 986-1031 | ATAAAGTATATGATATGTCAAAGCCTTTTTCATTCTTTGTTAGGCC | Entamoeba histolytica (AP023130.1) |
| Brisavirus isolate p48_v2_ET_w8_c3 (MZ405057.1) | 2069-2250 | ATTATTATCTTTGGTTCCCATTTTACGAACCCTCCCTTTATTTGCACAATGAGGGGATATCTGTCGAGTAAGCGTAACAAGAATGCCCATGTAGGCAATATATC | Entamoeba invadens (XM_004185684.1) |
| Brisavirus isolate p48_v2_ET_w8_c3 (MZ405057.1) | 986-1027 | ATAAAGTATATGATATGTCAAAGCCATTTTCCTTCTTTGTTA | Entamoeba histolytica (AP023130.1) |
| Vientovirus isolate p48_v2_ET_w11_c2 (MZ405066.1) | 2125-2297 | TACAAATCCTCCTTTCACTTGTACGATCAGGTTATAGCCATCAAGTAACCTGAGGAGGAAGCTCCATTCTGTAAGCATCGATTTCCTGAAGTCGTCAATGATTG | Entamoeba invadens (XM_004185684.1) |
| Vientovirus isolate p48_v2_ET_w11_c2 (MZ405066.1) | 986-1031 | ATAAAGTATATGATATGTCAAAGCCTTTTTCATTCTTTGTTAGGCC | Entamoeba histolytica (AP023130.1) |
| Vientovirus isolate p48_v2_ET_w10_c4 (MZ405064.1) | 2125-2297 | TACAAATCCTCCTTTCACTTGTACGATCAGGTTATAGCCATCAAGTAACCTGAGGAGGAAGCTCCATTCTGTAAGCATCGATTTCCTGAAGTCGTCAATGATTG | Entamoeba invadens (XM_004185684.1) |
| Vientovirus isolate p48_v2_ET_w10_c4 (MZ405064.1) | 986-1031 | ATAAAGTATATGATATGTCAAAGCCTTTTTCATTCTTTGTTAGGCC | Entamoeba histolytica (AP023130.1) |
| Vientovirus isolate p48_v2_ET_w9_c4 (MZ405062.1) | 2125-2297 | TACAAATCCTCCTTTCACTTGTACGATCAGGTTATAGCCATCAAGTAACCTGAGGAGGAAGCTCCATTCTGTAAGCATCGATTTCCTGAAGTCGTCAATGATTG | Entamoeba invadens (XM_004185684.1) |
| Vientovirus isolate p48_v2_ET_w9_c4 (MZ405062.1) | 986-1031 | ATAAAGTATATGATATGTCAAAGCCTTTTTCATTCTTTGTTAGGCC | Entamoeba histolytica (AP023130.1) |
| Vientovirus isolate p48_v1_ET_w5_c4 (MZ405050.1) | 2212-2289 | TCTGAAGTCATCAATGATGGCGATTTCTTGACCGTTGTAATTATCAAACCACTTTAAGGTTTCGTTCGAGATCCAATA | Entamoeba invadens (XM_004185684.1) |
| Vientovirus isolate p48_v1_ET_w5_c4 (MZ405050.1) | 986-1031 | ATAAAGTATATGATATGTCAAAGCCTTTTTCATTCTTTGTTAGGCC | Entamoeba histolytica (AP023130.1) |
| Vientovirus isolate p48_v1_ET_w4_c3 (MZ405046.1) | 2125-2297 | TACAAATCCTCCTTTCACTTGTACGATCAGGTTATAGCCATCAAGTAACCTGAGGAGGAAGCTCCATTCTGTAAGCATCGATTTCCTGAAGTCGTCAATGATTG | Entamoeba invadens (XM_004185684.1) |
| Vientovirus isolate p48_v1_ET_w4_c3 (MZ405046.1) | 986-1031 | ATAAAGTATATGATATGTCAAAGCCTTTTTCATTCTTTGTTAGGCC | Entamoeba histolytica (AP023130.1) |
| Vientovirus isolate p48_v1_ET_w2_c2 (MZ405041.1) | 2125-2297 | TACAAATCCTCCTTTCACTTGTACGATCAGGTTATAGCCATCAAGTAACCTGAGGAGGAAGCTCCATTCTGTAAGCATCGATTTCCTGAAGTCGTCAATGATTG | Entamoeba invadens (XM_004185684.1) |
| Vientovirus isolate p48_v1_ET_w2_c2 (MZ405041.1) | 986-1031 | ATAAAGTATATGATATGTCAAAGCCTTTTTCATTCTTTGTTAGGCC | Entamoeba histolytica (AP023130.1) |
| Vientovirus isolate p48_v1_ET_w1_c4 (MZ405038.1) | 2212-2289 | TCTGAAGTCATCAATGATGGCGATTTCTTGACCGTTGTAATTATCAAACCACTTTAAGGTTTCGTTCGAGATCCAATA | Entamoeba invadens (XM_004185684.1) |
| Vientovirus isolate p48_v1_ET_w1_c4 (MZ405038.1) | 986-1031 | ATAAAGTATATGATATGTCAAAGCCTTTTTCATTCTTTGTTAGGCC | Entamoeba histolytica (AP023130.1) |
| Vientovirus isolate ET738-12 (MZ405035.1) | 2212-2289 | TCTGAAGTCATCAATGATGGCGATTTCTTGACCGTTGTAATTATCAAACCACTTTAAGGTTTCGTTCGAGATCCAATA | Entamoeba invadens (XM_004185684.1) |
| Vientovirus isolate ET738-12 (MZ405035.1) | 986-1031 | ATAAAGTATATGATATGTCAAAGCCTTTTTCATTCTTTGTTAGGCC | Entamoeba histolytica (AP023130.1) |
| Vientovirus isolate ET203-9 (MZ405022.1) | 986-1027 | ATAAAGTATATGATATGTCAAAGCCTTTTTCATTCTTTGTTA | Entamoeba histolytica (AP023130.1) |
| Vientovirus isolate ET724-8 (MZ405033.1) | 2212-2289 | TCTGAAGTCATCAATTATTGCGAGCTTTTGACCGTGATATCCATCAAACCAACGTAAGGAGTCGTTTGATATCCAATAGTCTCCA | Entamoeba invadens (XM_004185684.1) |
| Vientovirus isolate ET724-8 (MZ405033.1) | 986-1027 | ATAAAGTATATGATATGTCAAAGCCTTTTTCATTCTTTGTTA | Entamoeba histolytica (AP023130.1) |
| Vientovirus isolate ET724-2 (MZ405030.1) | 2194-2278 | TCTGAAGTCATCAATTATTGCGAGCTTTTGACCGTGATATCCATCAAACCAACGTAAGGAGTCGTTTGATATCCAATAGTCTCCA | Entamoeba invadens (XM_004185684.1) |
| Vientovirus isolate ET724-2 (MZ405030.1) | 986-1027 | ATAAAGTATATGATATGTCAAAGCCTTTTTCATTCTTTGTTA | Entamoeba histolytica (AP023130.1) |
| Vientovirus isolate ET207-1 (MZ405023.1) | 986-1027 | ATAAAGTATATGATATGTCAAAGCCTTTTTCATTCTTTGTTA | Entamoeba histolytica (AP023130.1) |
| Vientovirus isolate CM895-9 (MZ405019.1) | 986-1027 | ATAAAGTATATGATATGTCAAAGCCTTTTTCATTCTTTGTTA | Entamoeba histolytica (AP023130.1) |

| query acc.ver | subject acc.ver | subject id. | subject length and type | % identity | alignment length | mismatches | gap opens | q. start | q. end | s. start | s. end | evalue | bit score |
|---|---|---|---|---|---|---|---|---|---|---|---|---|---|
| Vientovirus_MW_MK059772.1_pos2214-2301 | XM_004185684.1 | Entamoeba invadens IP1 replication-associated protein, putative (EIN_004330) mRNA, complete cds | 1,284 bp linear mRNA | 75,3 | 89 | 20 | 2 | 1 | 87 | 761 | 673 | 1,0E-07 | 55,4 |
| Vientovirus_MW_MK059772.1_pos986-1031 | AP023130.1 | Entamoeba histolytica HM-1:IMSS Clone 6 2001, chromosome 22 DNA, nearly complete genome | 467,248 bp linear DNA | 87,0 | 46 | 6 | 0 | 1 | 46 | 463656 | 463701 | 1,1E-08 | 57,2 |
| Vientovirus_XM_MK059771.1_pos1776-1830 | AP023127.1 | Entamoeba histolytica HM-1:IMSS Clone 6 2001, chromosome 19 DNA, nearly complete genome | 601,141 bp linear DNA | 82,9 | 41 | 7 | 0 | 13 | 53 | 74159 | 74119 | 3,3E-04 | 43,7 |
| Vientovirus_XM_MK059771.1_pos1776-1818 | AP023127.1 | Entamoeba histolytica HM-1:IMSS Clone 6 2001, chromosome 19 DNA, nearly complete genome | 601,141 bp linear DNA | 85,0 | 40 | 4 | 1 | 2 | 41 | 78101 | 78064 | 2,1E-04 | 43,7 |
| Vientovirus_XM_MK059771.1_pos1777-1818 | AP023127.1 | Entamoeba histolytica HM-1:IMSS Clone 6 2001, chromosome 19 DNA, nearly complete genome | 601,141 bp linear DNA | 85,0 | 40 | 4 | 1 | 1 | 40 | 78101 | 78064 | 2,0E-04 | 43,7 |
| Vientovirus_XM_MK059771.1_pos986-1027 | AP023130.1 | Entamoeba histolytica HM-1:IMSS Clone 6 2001, chromosome 22 DNA, nearly complete genome | 467,248 bp linear DNA | 88,1 | 42 | 5 | 0 | 1 | 42 | 463656 | 463697 | 1,1E-07 | 54,5 |
| Vientovirus_MC_MK059770.1_pos1770-1837 | AP023119.1 | Entamoeba histolytica HM-1:IMSS Clone 6 2001, chromosome 11 DNA, nearly complete genome | 1,194,977 bp linear DNA | 86,8 | 38 | 5 | 0 | 15 | 52 | 705576 | 705613 | 3,8E-05 | 47,3 |
| Vientovirus_MC_MK059770.1_pos986-1027 | AP023130.1 | Entamoeba histolytica HM-1:IMSS Clone 6 2001, chromosome 22 DNA, nearly complete genome | 467,248 bp linear DNA | 88,1 | 42 | 5 | 0 | 1 | 42 | 463656 | 463697 | 1,1E-07 | 54,5 |
| Vientovirus_LZ_MK059769.1_pos2210-2294 | XM_004185684.1 | Entamoeba invadens IP1 replication-associated protein, putative (EIN_004330) mRNA, complete cds | 1,284 bp linear mRNA | 80,2 | 86 | 15 | 2 | 1 | 85 | 762 | 678 | 1,3E-12 | 72,5 |
| Vientovirus_LZ_MK059769.1_pos | XM_004185684.1 | Entamoeba invadens IP1 replication-associated protein, putative (EIN_004330) mRNA, complete cds | 1,284 bp linear mRNA | 80,2 | 86 | 15 | 2 | 1 | 85 | 762 | 678 | 1,3E-12 | 72,5 |
| Vientovirus_LZ_MK059769.1_pos986-1031 | AP023130.1 | Entamoeba histolytica HM-1:IMSS Clone 6 2001, chromosome 22 DNA, nearly complete genome | 467,248 bp linear DNA | 87,0 | 46 | 6 | 0 | 1 | 46 | 463656 | 463701 | 1,1E-08 | 57,2 |
| Vientovirus_EC_MK059768.1_pos1768-1822 | AP023127.1 | Entamoeba histolytica HM-1:IMSS Clone 6 2001, chromosome 19 DNA, nearly complete genome | 601,141 bp linear DNA | 78,6 | 56 | 9 | 2 | 1 | 55 | 96089 | 96036 | 1,0E-03 | 41,9 |
| Vientovirus_EC_MK059768.1_pos1772-1826 | AP023135.1 | Entamoeba histolytica HM-1:IMSS Clone 6 2001, chromosome 27 DNA, nearly complete genome | 118,222 bp linear DNA | 95,8 | 24 | 1 | 0 | 18 | 41 | 62487 | 62464 | 4,0E-03 | 40,1 |
| Vientovirus_EC_MK059768.1_pos1796-1832 | AP023115.1 | Entamoeba histolytica HM-1:IMSS Clone 6 2001, chromosome 7 DNA, nearly complete genome | 1,398,980 bp linear DNA | 84,8 | 33 | 5 | 0 | 4 | 36 | 188562 | 188594 | 6,0E-03 | 38,3 |
| Vientovirus_EC_MK059768.1_pos986-1031 | AP023130.1 | Entamoeba histolytica HM-1:IMSS Clone 6 2001, chromosome 22 DNA, nearly complete genome | 467,248 bp linear DNA | 87,0 | 46 | 6 | 0 | 1 | 46 | 463656 | 463701 | 1,1E-08 | 57,2 |
| Vientovirus_AV_MK059767.1_pos986-1031 | AP023130.1 | Entamoeba histolytica HM-1:IMSS Clone 6 2001, chromosome 22 DNA, nearly complete genome | 467,248 bp linear DNA | 87,0 | 46 | 6 | 0 | 1 | 46 | 463656 | 463701 | 1,1E-08 | 57,2 |
| Vientovirus_FB_MK059763.1_pos2125-2297 | XM_004185684.1 | Entamoeba invadens IP1 replication-associated protein, putative (EIN_004330) mRNA, complete cds | 1,284 bp linear mRNA | 70,1 | 174 | 48 | 3 | 1 | 173 | 843 | 673 | 1,1E-11 | 70,7 |
| Vientovirus_FB_MK059763.1_pos986-1031 | AP023130.1 | Entamoeba histolytica HM-1:IMSS Clone 6 2001, chromosome 22 DNA, nearly complete genome | 467,248 bp linear DNA | 87,0 | 46 | 6 | 0 | 1 | 46 | 463656 | 463701 | 1,1E-08 | 57,2 |
| Vientovirus_ES_MK059762.1_pos2449-2531 | XM_008856450.1 | Entamoeba nuttalli P19 protein kinase domain containing protein partial mRNA | 2,736 bp linear mRNA | 85,3 | 34 | 3 | 1 | 7 | 38 | 2242 | 2209 | 2,6E-02 | 37,4 |
| Vientovirus_ES_MK059762.1_pos986-1031 | AP023130.1 | Entamoeba histolytica HM-1:IMSS Clone 6 2001, chromosome 22 DNA, nearly complete genome | 467,248 bp linear DNA | 87,0 | 46 | 6 | 0 | 1 | 46 | 463656 | 463701 | 1,1E-08 | 57,2 |
| Vientovirus_DC_MK059761.1_pos2122-2294 | XM_004185684.1 | Entamoeba invadens IP1 replication-associated protein, putative (EIN_004330) mRNA, complete cds | 1,284 bp linear mRNA | 70,1 | 174 | 48 | 3 | 1 | 173 | 843 | 673 | 1,1E-11 | 70,7 |
| Vientovirus_DC_MK059761.1_pos986-1031 | AP023130.1 | Entamoeba histolytica HM-1:IMSS Clone 6 2001, chromosome 22 DNA, nearly complete genome | 467,248 bp linear DNA | 87,0 | 46 | 6 | 0 | 1 | 46 | 463656 | 463701 | 1,1E-08 | 57,2 |
| Vientovirus_VN_MT759843.1_pos2207-2284 | XM_004185684.1 | Entamoeba invadens IP1 replication-associated protein, putative (EIN_004330) mRNA, complete cds | 1,284 bp linear mRNA | 78,5 | 79 | 15 | 2 | 1 | 78 | 762 | 685 | 7,3E-09 | 59,9 |
| Vientovirus_VN_MT759843.1_pos2937-3001 | AP023113.1 | Entamoeba histolytica HM-1:IMSS Clone 6 2001, chromosome 5 DNA, nearly complete genome | 1,590,735 bp linear DNA | 78,2 | 55 | 11 | 1 | 7 | 61 | 1523604 | 1523551 | 4,3E-04 | 42,8 |
| Vientovirus_VN_MT759843.1_pos986-1031 | AP023130.1 | Entamoeba histolytica HM-1:IMSS Clone 6 2001, chromosome 22 DNA, nearly complete genome | 467,248 bp linear DNA | 87,0 | 46 | 6 | 0 | 1 | 46 | 463656 | 463701 | 1,1E-08 | 57,2 |
| Vientovirus_JB_MK059764.1_pos986-1031 | AP023130.1 | Entamoeba histolytica HM-1:IMSS Clone 6 2001, chromosome 22 DNA, nearly complete genome | 467,248 bp linear DNA | 87,0 | 46 | 6 | 0 | 1 | 46 | 463656 | 463701 | 1,1E-08 | 57,2 |
| Brisavirus_II_MK059755.1_pos1804-1881 | AP023111.1 | Entamoeba histolytica HM-1:IMSS Clone 6 2001, chromosome 3 DNA, nearly complete genome | 1,619,944 bp linear DNA | 78,3 | 60 | 11 | 1 | 1 | 58 | 518483 | 518424 | 1,3E-05 | 48,2 |
| Brisavirus_II_MK059755.1_pos1828-1866 | XM_008861204.1 | Entamoeba nuttalli P19 leucine rich repeat protein, BspA family protein partial mRNA | 660 bp linear mRNA | 92,9 | 28 | 1 | 1 | 9 | 36 | 49 | 75 | 2,0E-03 | 39,2 |
| Brisavirus_II_MK059755.1_pos1764-1854 | AP023119.1 | Entamoeba histolytica HM-1:IMSS Clone 6 2001, chromosome 11 DNA, nearly complete genome | 1,194,977 bp linear DNA | 78,4 | 51 | 11 | 0 | 38 | 88 | 898525 | 898575 | 7,0E-04 | 43,7 |
| Brisavirus_II_MK059755.1_pos986-1031 | AP023130.1 | Entamoeba histolytica HM-1:IMSS Clone 6 2001, chromosome 22 DNA, nearly complete genome | 467,248 bp linear DNA | 87,0 | 46 | 6 | 0 | 1 | 46 | 463656 | 463701 | 1,1E-08 | 57,2 |
| Brisavirus_MD_MK059756.1_pos1111-1167 | AP023129.1 | Entamoeba histolytica HM-1:IMSS Clone 6 2001, chromosome 21 DNA, nearly complete genome | 476,802 bp linear DNA | 87,9 | 33 | 3 | 1 | 26 | 57 | 213738 | 213770 | 4,0E-03 | 39,2 |
| Brisavirus_VW_MK059759.1_pos2453-2508 | XM_001733488.1 | Entamoeba dispar SAW760 hypothetical protein EDI_164160 mRNA, complete cds | 636 bp linear mRNA | 88,2 | 34 | 4 | 0 | 21 | 54 | 436 | 403 | 9,7E-05 | 44,6 |
| Brisavirus_VW_MK059759.1_pos1773-1824 | AP023111.1 | Entamoeba histolytica HM-1:IMSS Clone 6 2001, chromosome 3 DNA, nearly complete genome | 1,619,944 bp linear DNA | 96,0 | 25 | 1 | 0 | 8 | 32 | 765754 | 765730 | 1,0E-03 | 41,9 |
| Brisavirus_RC_MK059757.1_pos2504-2577 | XM_008861037.1 | Entamoeba nuttalli P19 Spc97 / Spc98 family protein partial mRNA | 1,908 bp linear mRNA | 92,0 | 25 | 2 | 0 | 32 | 56 | 985 | 961 | 2,2E-02 | 37,4 |
| Brisavirus_RC_MK059757.1_pos986-1027 | AP023130.1 | Entamoeba histolytica HM-1:IMSS Clone 6 2001, chromosome 22 DNA, nearly complete genome | 467,248 bp linear DNA | 88,1 | 42 | 5 | 0 | 1 | 42 | 463656 | 463697 | 1,1E-07 | 54,5 |
| Brisavirus_YH_MK059758.1_pos2061-2242 | XM_004185684.1 | Entamoeba invadens IP1 replication-associated protein, putative (EIN_004330) mRNA, complete cds | 1,284 bp linear mRNA | 68,1 | 182 | 55 | 2 | 1 | 182 | 866 | 688 | 6,2E-09 | 61,7 |
| Brisavirus_AA_MK059754.1_pos986-1031 | AP023130.1 | Entamoeba histolytica HM-1:IMSS Clone 6 2001, chromosome 22 DNA, nearly complete genome | 467,248 bp linear DNA | 87,0 | 46 | 6 | 0 | 1 | 46 | 463656 | 463701 | 1,1E-08 | 57,2 |
| Human_respiratory_circular_DNA_virus_isolate_15232_KY328746.1_pos2211-2295 | XM_004185684.1 | Entamoeba invadens IP1 replication-associated protein, putative (EIN_004330) mRNA, complete cds | 1,284 bp linear mRNA | 80,2 | 86 | 15 | 2 | 1 | 85 | 762 | 678 | 1,3E-12 | 72,5 |
| Human_respiratory_circular_DNA_virus_isolate_15232_KY328746.1_pos986-1031 | AP023130.1 | Entamoeba histolytica HM-1:IMSS Clone 6 2001, chromosome 22 DNA, nearly complete genome | 467,248 bp linear DNA | 87,0 | 46 | 6 | 0 | 1 | 46 | 463656 | 463701 | 1,1E-08 | 57,2 |
| Human_respiratory_circular_DNA_virus_isolate_15037_KY328745.1_pos2211-2295 | XM_004185684.1 | Entamoeba invadens IP1 replication-associated protein, putative (EIN_004330) mRNA, complete cds | 1,284 bp linear mRNA | 80,2 | 86 | 15 | 2 | 1 | 85 | 762 | 678 | 1,3E-12 | 72,5 |
| Human_respiratory_circular_DNA_virus_isolate_15037_KY328745.1_pos986-1031 | AP023130.1 | Entamoeba histolytica HM-1:IMSS Clone 6 2001, chromosome 22 DNA, nearly complete genome | 467,248 bp linear DNA | 87,0 | 46 | 6 | 0 | 1 | 46 | 463656 | 463701 | 1,1E-08 | 57,2 |
| Human_respiratory_circular_DNA_virus_isolate_15040_KY244146.1,_15065_KY579361.1,_15027_KY579360.1,_15078_KY579362.1_pos2211-2295 | XM_004185684.1 | Entamoeba invadens IP1 replication-associated protein, putative (EIN_004330) mRNA, complete cds | 1,284 bp linear mRNA | 80,2 | 86 | 15 | 2 | 1 | 85 | 762 | 678 | 1,3E-12 | 72,5 |
| Human_respiratory_circular_DNA_virus_isolate_15040_KY244146.1,_15065_KY579361.1,_15027_KY579360.1,_15078_KY579362.1_pos986-1031 | AP023130.1 | Entamoeba histolytica HM-1:IMSS Clone 6 2001, chromosome 22 DNA, nearly complete genome | 467,248 bp linear DNA | 87,0 | 46 | 6 | 0 | 1 | 46 | 463656 | 463701 | 1,1E-08 | 57,2 |
| Redondovirus_sp._Isolate_1_MT482428.1_pos989-1030 | AP023130.1 | Entamoeba histolytica HM-1:IMSS Clone 6 2001, chromosome 22 DNA, nearly complete genome | 467,248 bp linear DNA | 88,1 | 42 | 5 | 0 | 1 | 42 | 463656 | 463697 | 1,1E-07 | 54,5 |
| Redondovirus_sp._Isolate_10_MT482429.1_pos2203-2277 | XM_004185684.1 | Entamoeba invadens IP1 replication-associated protein, putative (EIN_004330) mRNA, complete cds | 1,284 bp linear mRNA | 76,0 | 75 | 18 | 0 | 1 | 75 | 755 | 681 | 8,4E-08 | 55,4 |
| Redondovirus_sp._Isolate_10_MT482429.1_pos986-1027 | AP023130.1 | Entamoeba histolytica HM-1:IMSS Clone 6 2001, chromosome 22 DNA, nearly complete genome | 467,248 bp linear DNA | 88,1 | 42 | 5 | 0 | 1 | 42 | 463656 | 463697 | 1,1E-07 | 54,5 |
| Redondovirus_sp._Isolate_11_MT482430.1_pos2125-2297 | XM_004185684.1 | Entamoeba invadens IP1 replication-associated protein, putative (EIN_004330) mRNA, complete cds | 1,284 bp linear mRNA | 70,7 | 174 | 47 | 3 | 1 | 173 | 843 | 673 | 2,6E-13 | 75,2 |
| Redondovirus_sp._Isolate_11_MT482430.1_pos986-1031 | AP023130.1 | Entamoeba histolytica HM-1:IMSS Clone 6 2001, chromosome 22 DNA, nearly complete genome | 467,248 bp linear DNA | 87,0 | 46 | 6 | 0 | 1 | 46 | 463656 | 463701 | 1,1E-08 | 57,2 |
| Redondovirus_sp._Isolate_25_MT482431.1_pos986-1027 | AP023130.1 | Entamoeba histolytica HM-1:IMSS Clone 6 2001, chromosome 22 DNA, nearly complete genome | 467,248 bp linear DNA | 88,1 | 42 | 5 | 0 | 1 | 42 | 463656 | 463697 | 1,1E-07 | 54,5 |
| Redondovirus_sp._Isolate_26_MT482432.1_pos986-1027 | AP023130.1 | Entamoeba histolytica HM-1:IMSS Clone 6 2001, chromosome 22 DNA, nearly complete genome | 467,248 bp linear DNA | 88,1 | 42 | 5 | 0 | 1 | 42 | 463656 | 463697 | 1,1E-07 | 54,5 |
| Vientovirus_isolate_p67_20161228_ET_WGA_B_MZ405079.1_pos2921-2975 | AP023113.1 | Entamoeba histolytica HM-1:IMSS Clone 6 2001, chromosome 5 DNA, nearly complete genome | 1,590,735 bp linear DNA | 81,1 | 37 | 7 | 0 | 19 | 55 | 1523604 | 1523568 | 4,9E-02 | 36,5 |
| Vientovirus_isolate_p67_20161228_ET_WGA_B_MZ405079.1_pos986-1031 | AP023130.1 | Entamoeba histolytica HM-1:IMSS Clone 6 2001, chromosome 22 DNA, nearly complete genome | 467,248 bp linear DNA | 87,0 | 46 | 6 | 0 | 1 | 46 | 463656 | 463701 | 1,1E-08 | 57,2 |
| Vientovirus_isolate_p67_20161228_ET_WGA_A_MZ405078.1_pos2208-2292 | XM_004185684.1 | Entamoeba invadens IP1 replication-associated protein, putative (EIN_004330) mRNA, complete cds | 1,284 bp linear mRNA | 80,2 | 86 | 15 | 2 | 1 | 85 | 762 | 678 | 1,3E-12 | 72,5 |
| Vientovirus_isolate_p67_20161228_ET_WGA_A_MZ405078.1_pos2922-2976 | AP023113.1 | Entamoeba histolytica HM-1:IMSS Clone 6 2001, chromosome 5 DNA, nearly complete genome | 1,590,735 bp linear DNA | 81,1 | 37 | 7 | 0 | 19 | 55 | 1523604 | 1523568 | 4,9E-02 | 36,5 |

| query acc.ver | subject acc.ver | subject id. | subject length and type | % identity | alignment length | mismatches | gap opens | q. start | q. end | s. start | s. end | evalue | bit score |
|---|---|---|---|---|---|---|---|---|---|---|---|---|---|
| Vientovirus_isolate_p67_20161228_ET_WGA_A_MZ405078.1_pos986-1031 | AP023130.1 | Entamoeba histolytica HM-1:IMSS Clone 6 2001, chromosome 22 DNA, nearly complete genome | 467,248 bp linear DNA | 87,0 | 46 | 6 | 0 | 1 | 46 | 463656 | 463701 | 1,1E-08 | 57,2 |
| Vientovirus_isolate_p67_20161223_ET_A1_MZ405077.1_pos986-1031 | AP023130.1 | Entamoeba histolytica HM-1:IMSS Clone 6 2001, chromosome 22 DNA, nearly complete genome | 467,248 bp linear DNA | 87,0 | 46 | 6 | 0 | 1 | 46 | 463656 | 463701 | 1,1E-08 | 57,2 |
| Vientovirus_isolate_p67_20161216_ET_C1_MZ405076.1_pos2921-2975 | AP023113.1 | Entamoeba histolytica HM-1:IMSS Clone 6 2001, chromosome 5 DNA, nearly complete genome | 1,590,735 bp linear DNA | 81,1 | 37 | 7 | 0 | 19 | 55 | 1523604 | 1523568 | 4,9E-02 | 36,5 |
| Vientovirus_isolate_p67_20161216_ET_C1_MZ405076.1_pos986-1031 | AP023130.1 | Entamoeba histolytica HM-1:IMSS Clone 6 2001, chromosome 22 DNA, nearly complete genome | 467,248 bp linear DNA | 87,0 | 46 | 6 | 0 | 1 | 46 | 463656 | 463701 | 1,1E-08 | 57,2 |
| Vientovirus_isolate_p67_20161216_ET_B10_MZ405075.1_pos2918-2972 | AP023113.1 | Entamoeba histolytica HM-1:IMSS Clone 6 2001, chromosome 5 DNA, nearly complete genome | 1,590,735 bp linear DNA | 81,1 | 37 | 7 | 0 | 19 | 55 | 1523604 | 1523568 | 4,9E-02 | 36,5 |
| Vientovirus_isolate_p67_20161216_ET_B10_MZ405075.1_pos986-1031 | AP023130.1 | Entamoeba histolytica HM-1:IMSS Clone 6 2001, chromosome 22 DNA, nearly complete genome | 467,248 bp linear DNA | 87,0 | 46 | 6 | 0 | 1 | 46 | 463656 | 463701 | 1,1E-08 | 57,2 |
| Brisavirus_isolate_p67_20161208_OP_D7_MZ405073.1_pos317-370 | XM_004255428.1 | Entamoeba invadens IP1 hypothetical protein (EIN_194640) mRNA, complete cds | 792 bp linear mRNA | 100,0 | 19 | 0 | 0 | 17 | 35 | 281 | 299 | 4,7E-02 | 35,6 |
| Brisavirus_isolate_p67_20161208_OP_D7_MZ405073.1_pos986-1031 | AP023130.1 | Entamoeba histolytica HM-1:IMSS Clone 6 2001, chromosome 22 DNA, nearly complete genome | 467,248 bp linear DNA | 87,0 | 46 | 6 | 0 | 1 | 46 | 463656 | 463701 | 1,1E-08 | 57,2 |
| Brisavirus_isolate_p48_v2_ET_w8_c3_MZ405057.1_pos2069-2250 | XM_004185684.1 | Entamoeba invadens IP1 replication-associated protein, putative (EIN_004330) mRNA, complete cds | 1,284 bp linear mRNA | 68,7 | 182 | 54 | 2 | 1 | 182 | 866 | 688 | 1,5E-10 | 66,2 |
| Brisavirus_isolate_p48_v2_ET_w8_c3_MZ405057.1_pos986-1027 | AP023130.1 | Entamoeba histolytica HM-1:IMSS Clone 6 2001, chromosome 22 DNA, nearly complete genome | 467,248 bp linear DNA | 88,1 | 42 | 5 | 0 | 1 | 42 | 463656 | 463697 | 1,1E-07 | 54,5 |
| Vientovirus_isolate_p48_v2_ET_w11_c2_MZ405066.1_pos2125-2297 | XM_004185684.1 | Entamoeba invadens IP1 replication-associated protein, putative (EIN_004330) mRNA, complete cds | 1,284 bp linear mRNA | 70,1 | 174 | 48 | 3 | 1 | 173 | 843 | 673 | 1,1E-11 | 70,7 |
| Vientovirus_isolate_p48_v2_ET_w11_c2_MZ405066.1_pos986-1031 | AP023130.1 | Entamoeba histolytica HM-1:IMSS Clone 6 2001, chromosome 22 DNA, nearly complete genome | 467,248 bp linear DNA | 87,0 | 46 | 6 | 0 | 1 | 46 | 463656 | 463701 | 1,1E-08 | 57,2 |
| Vientovirus_isolate_p48_v2_ET_w10_c4_MZ405064.1_pos2125-2297 | XM_004185684.1 | Entamoeba invadens IP1 replication-associated protein, putative (EIN_004330) mRNA, complete cds | 1,284 bp linear mRNA | 70,1 | 174 | 48 | 3 | 1 | 173 | 843 | 673 | 1,1E-11 | 70,7 |
| Vientovirus_isolate_p48_v2_ET_w10_c4_MZ405064.1_pos986-1031 | AP023130.1 | Entamoeba histolytica HM-1:IMSS Clone 6 2001, chromosome 22 DNA, nearly complete genome | 467,248 bp linear DNA | 87,0 | 46 | 6 | 0 | 1 | 46 | 463656 | 463701 | 1,1E-08 | 57,2 |
| Vientovirus_isolate_p48_v2_ET_w9_c4_MZ405062.1_pos2125-2297 | XM_004185684.1 | Entamoeba invadens IP1 replication-associated protein, putative (EIN_004330) mRNA, complete cds | 1,284 bp linear mRNA | 70,1 | 174 | 48 | 3 | 1 | 173 | 843 | 673 | 1,1E-11 | 70,7 |
| Vientovirus_isolate_p48_v2_ET_w9_c4_MZ405062.1_pos986-1031 | AP023130.1 | Entamoeba histolytica HM-1:IMSS Clone 6 2001, chromosome 22 DNA, nearly complete genome | 467,248 bp linear DNA | 87,0 | 46 | 6 | 0 | 1 | 46 | 463656 | 463701 | 1,1E-08 | 57,2 |
| Vientovirus_isolate_p48_v1_ET_w5_c4_MZ405050.1_pos2212-2289 | XM_004185684.1 | Entamoeba invadens IP1 replication-associated protein, putative (EIN_004330) mRNA, complete cds | 1,284 bp linear mRNA | 78,5 | 79 | 15 | 2 | 1 | 78 | 762 | 685 | 7,3E-09 | 59,9 |
| Vientovirus_isolate_p48_v1_ET_w5_c4_MZ405050.1_pos986-1031 | AP023130.1 | Entamoeba histolytica HM-1:IMSS Clone 6 2001, chromosome 22 DNA, nearly complete genome | 467,248 bp linear DNA | 87,0 | 46 | 6 | 0 | 1 | 46 | 463656 | 463701 | 1,1E-08 | 57,2 |
| Vientovirus_isolate_p48_v1_ET_w4_c3_MZ405046.1_pos2125-2297 | XM_004185684.1 | Entamoeba invadens IP1 replication-associated protein, putative (EIN_004330) mRNA, complete cds | 1,284 bp linear mRNA | 70,1 | 174 | 48 | 3 | 1 | 173 | 843 | 673 | 1,1E-11 | 70,7 |
| Vientovirus_isolate_p48_v1_ET_w4_c3_MZ405046.1_pos986-1031 | AP023130.1 | Entamoeba histolytica HM-1:IMSS Clone 6 2001, chromosome 22 DNA, nearly complete genome | 467,248 bp linear DNA | 87,0 | 46 | 6 | 0 | 1 | 46 | 463656 | 463701 | 1,1E-08 | 57,2 |
| Vientovirus_isolate_p48_v1_ET_w2_c2_MZ405041.1_pos2125-2297 | XM_004185684.1 | Entamoeba invadens IP1 replication-associated protein, putative (EIN_004330) mRNA, complete cds | 1,284 bp linear mRNA | 70,1 | 174 | 48 | 3 | 1 | 173 | 843 | 673 | 1,1E-11 | 70,7 |
| Vientovirus_isolate_p48_v1_ET_w2_c2_MZ405041.1_pos986-1031 | AP023130.1 | Entamoeba histolytica HM-1:IMSS Clone 6 2001, chromosome 22 DNA, nearly complete genome | 467,248 bp linear DNA | 87,0 | 46 | 6 | 0 | 1 | 46 | 463656 | 463701 | 1,1E-08 | 57,2 |
| Vientovirus_isolate_p48_v1_ET_w1_c4_MZ405038.1_pos2212-2289 | XM_004185684.1 | Entamoeba invadens IP1 replication-associated protein, putative (EIN_004330) mRNA, complete cds | 1,284 bp linear mRNA | 78,5 | 79 | 15 | 2 | 1 | 78 | 762 | 685 | 7,3E-09 | 59,9 |
| Vientovirus_isolate_p48_v1_ET_w1_c4_MZ405038.1_pos986-1031 | AP023130.1 | Entamoeba histolytica HM-1:IMSS Clone 6 2001, chromosome 22 DNA, nearly complete genome | 467,248 bp linear DNA | 87,0 | 46 | 6 | 0 | 1 | 46 | 463656 | 463701 | 1,1E-08 | 57,2 |
| Vientovirus_isolate_ET738-12_MZ405035.1_pos2212-2289 | XM_004185684.1 | Entamoeba invadens IP1 replication-associated protein, putative (EIN_004330) mRNA, complete cds | 1,284 bp linear mRNA | 78,5 | 79 | 15 | 2 | 1 | 78 | 762 | 685 | 7,3E-09 | 59,9 |
| Vientovirus_isolate_ET738-12_MZ405035.1_pos986-1031 | AP023130.1 | Entamoeba histolytica HM-1:IMSS Clone 6 2001, chromosome 22 DNA, nearly complete genome | 467,248 bp linear DNA | 87,0 | 46 | 6 | 0 | 1 | 46 | 463656 | 463701 | 1,1E-08 | 57,2 |
| Vientovirus_isolate_ET203-9_MZ405022.1_pos986-1027 | AP023130.1 | Entamoeba histolytica HM-1:IMSS Clone 6 2001, chromosome 22 DNA, nearly complete genome | 467,248 bp linear DNA | 88,1 | 42 | 5 | 0 | 1 | 42 | 463656 | 463697 | 1,1E-07 | 54,5 |
| Vientovirus_isolate_ET724-8_MZ405033.1_pos2212-2289 | XM_004185684.1 | Entamoeba invadens IP1 replication-associated protein, putative (EIN_004330) mRNA, complete cds | 1,284 bp linear mRNA | 80,2 | 86 | 15 | 2 | 1 | 85 | 762 | 678 | 1,3E-12 | 72,5 |
| Vientovirus_isolate_ET724-8_MZ405033.1_pos986-1027 | AP023130.1 | Entamoeba histolytica HM-1:IMSS Clone 6 2001, chromosome 22 DNA, nearly complete genome | 467,248 bp linear DNA | 88,1 | 42 | 5 | 0 | 1 | 42 | 463656 | 463697 | 1,1E-07 | 54,5 |
| Vientovirus_isolate_ET724-2_MZ405030.1_pos2194-2278 | XM_004185684.1 | Entamoeba invadens IP1 replication-associated protein, putative (EIN_004330) mRNA, complete cds | 1,284 bp linear mRNA | 80,2 | 86 | 15 | 2 | 1 | 85 | 762 | 678 | 1,3E-12 | 72,5 |
| Vientovirus_isolate_ET724-2_MZ405030.1_pos1771-1837 | AP023119.1 | Entamoeba histolytica HM-1:IMSS Clone 6 2001, chromosome 11 DNA, nearly complete genome | 1,194,977 bp linear DNA | 86,8 | 38 | 4 | 1 | 15 | 51 | 705576 | 705613 | 4,6E-04 | 43,7 |
| Vientovirus_isolate_ET724-2_MZ405030.1_pos986-1027 | AP023130.1 | Entamoeba histolytica HM-1:IMSS Clone 6 2001, chromosome 22 DNA, nearly complete genome | 467,248 bp linear DNA | 88,1 | 42 | 5 | 0 | 1 | 42 | 463656 | 463697 | 1,1E-07 | 54,5 |
| Vientovirus_isolate_ET207-1_MZ405023.1_pos986-1027 | AP023130.1 | Entamoeba histolytica HM-1:IMSS Clone 6 2001, chromosome 22 DNA, nearly complete genome | 467,248 bp linear DNA | 88,1 | 42 | 5 | 0 | 1 | 42 | 463656 | 463697 | 1,1E-07 | 54,5 |
| Vientovirus_isolate_CM895-9_MZ405019.1_pos986-1027 | AP023130.1 | Entamoeba histolytica HM-1:IMSS Clone 6 2001, chromosome 22 DNA, nearly complete genome | 467,248 bp linear DNA | 88,1 | 42 | 5 | 0 | 1 | 42 | 463656 | 463697 | 1,1E-07 | 54,5 |
| Brisavirus_isolate_ET239-2_MZ405025.1_pos1795-1848 | XM_008858086.1 | Entamoeba nuttalli P19 RNA pseudouridine synthase superfamily protein partial mRNA | 1,209 bp linear mRNA | 86,8 | 38 | 2 | 2 | 2 | 39 | 301 | 335 | 1,0E-03 | 41,0 |
| Brisavirus_isolate_ET239-2_MZ405025.1_pos2443-2502 | AP023126.1 | Entamoeba histolytica HM-1:IMSS Clone 6 2001, chromosome 18 DNA, nearly complete genome | 732,483 bp linear DNA | 84,0 | 50 | 6 | 2 | 7 | 54 | 297379 | 297330 | 9,0E-06 | 48,2 |
| Brisavirus_isolate_ET239-2_MZ405025.1_pos1785-1846 | XM_008860033.1 | Entamoeba nuttalli P19 Sec6 protein, putative partial mRNA | 2,301 bp linear mRNA | 92,6 | 27 | 2 | 0 | 9 | 35 | 358 | 384 | 1,0E-03 | 41,0 |
